# Nucleosome Assembly Proteins Get SET to Defeat the Guardian of Chromosome Cohesion

**DOI:** 10.1371/journal.pgen.1003829

**Published:** 2013-09-26

**Authors:** Jonathan M. G. Higgins, Mary Herbert

**Affiliations:** 1 Division of Rheumatology, Immunology and Allergy, Brigham and Women's Hospital, Harvard Medical School, Boston, Massachusetts, United States of America; 2 Newcastle Fertility Centre and Institute for Aging and Health, International Centre for Life, Newcastle University, Newcastle upon Tyne, United Kingdom; Duke University, United States of America

Cohesion between sister chromosomes is a critical mechanism used by eukaryotic cells to accomplish accurate chromosome segregation. As an analogy, imagine that you are struck one day by the (inexplicable) urge to segregate all your socks into two equal piles. The task will be much easier if you previously took the time to pair them up before tossing them in your dresser drawer. Similarly, keeping sister chromosomes together following DNA replication allows them to be efficiently sorted during cell division. In mitosis, cohesion at centromeres promotes bi-orientation of sister kinetochores by counteracting the pulling forces of microtubules emanating from opposite spindle poles. In meiosis, cohesion between chromosome arms facilitates segregation of recombined homologues during meiosis I by stabilizing the physical linkages (chiasmata) between them, and cohesion between centromeres is essential for accurate segregation of sisters in meiosis II [1], [2]. A study by Moshkin and colleagues in this issue of *PLOS Genetics* [3] sheds new light on how these processes are regulated.

**Figure 1:**
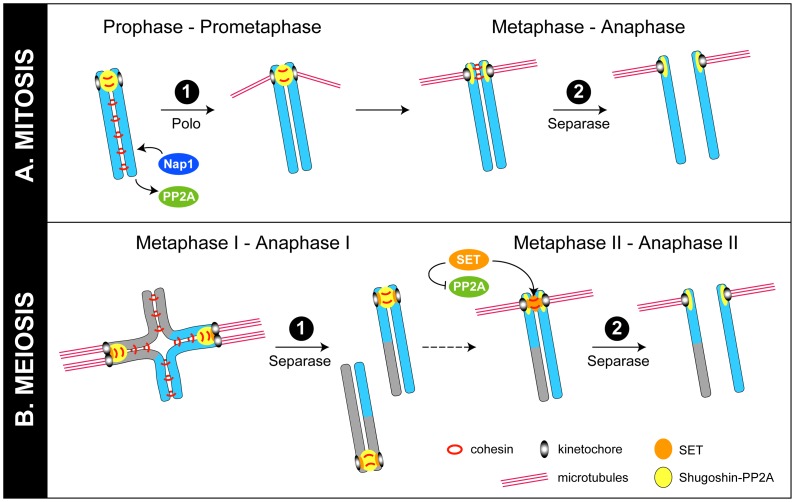
Models for regulation of chromosome cohesion in mitosis and meiosis. (A) In mitosis, cohesin (red rings) is phosphorylated and removed from chromosome arms by the “prophase pathway.” In this issue, Moshkin et al. provide evidence that Nap1 (blue) can displace PP2A (green) from cohesin to further promote cohesin release from chromosomes [3]. Cohesin at centromeres is protected by shugoshin-PP2A (yellow) until the metaphase–anaphase transition, when tension across bi-oriented sister kinetochores leads to the movement of shugoshin-PP2A away from cohesin at inner centromeres, allowing cohesin to be cleaved by separase [6]. It is not known if Nap1 has a role at this stage. (B) In meiosis I, phosphorylated cohesin linking sister chromosome arms is cleaved by separase, allowing recombined homologues to segregate. Cohesin at centromeres, however, is protected by shugoshin-PP2A, so that sister chromosomes remain together. In meiosis II, as in mitosis, the movement of shugoshin-PP2A away from inner centromeres on bi-oriented chromosomes allows cohesin between sisters to be phosphorylated and cleaved by separase [5], [6]. Recent work from Chambon et al. and Qi et al. is consistent with the view that the Nap1-related protein SET (orange) relocates to inner centromeres in meiosis II to inhibit PP2A and provide an additional means to encourage cohesin phosphorylation and cleavage by separase [8], [9].

Cohesion is brought about by ring-shaped cohesin complexes, which contain Smc1, Smc3, a kleisin (mainly Rad21/Scc1in mitosis and Rec8 in meiosis), and an associated SA/Scc3 subunit. In many animals, cohesion removal in mitosis occurs in two steps (Figure 1A). First, in the “prophase pathway,” phosphorylation of SA by kinases such as Polo triggers non-proteolytic removal of cohesin from chromosome arms. This promotes removal of the bulk of cohesin from the arms but, importantly, does not dissolve cohesion at centromeres. Later, once chromosomes are bi-oriented and the spindle checkpoint is satisfied, a proteolytic cohesion removal system is let loose: Separase cleaves the Rad21/Scc1 subunit of the remaining chromosome-bound cohesin, triggering chromosome separation and allowing anaphase [1]. In meiosis, removal of cohesin also occurs by a two-step process but, in contrast to mitosis, both steps require separase activity (Figure 1B). During meiosis I, separase cleaves Rec8 on the arms, leading to resolution of chiasmata and disjunction of homologues. Rec8 at centromeres is not cleaved until meiosis II, when the sisters separate, finally giving rise to a haploid gamete [1], [2].

Although the two-step removal systems in mitosis and meiosis are distinct, a common protein complex is implicated in protecting centromeric cohesion during the first step in both cases. Shugoshin/MEI-S332 family proteins collaborate with the phosphatase PP2A to prevent cohesin removal at centromeres [2], [4]. In mitosis, shugoshin-PP2A complexes antagonize SA phosphorylation by mitotic kinases, preventing removal by the prophase pathway (Figure 1A). In meiosis, shugoshin-PP2A antagonizes phosphorylation of Rec8, preventing cleavage by separase (Figure 1B) [2], [4]. A key question has been: What subsequently allows centromeric cohesion to be cleaved by separase in the second step? One proposed model is that, in response to tension across bi-oriented sister kinetochores, shugoshin-PP2A complexes move away from cohesin complexes at inner centromeres, making cohesin susceptible to removal by separase [5], [6]. Newly published studies, described below, propose two additional (related) mechanisms that target PP2A to make cohesin sensitive to removal.

Chambon et al. suggest that an inhibitor of PP2A, known as SET (or I2PP2A or TAF-I) [7], is required to inactivate shugoshin-PP2A [8]. They reported, as in previous proteomic studies, that SET is found in a complex with shugoshin, and that it more clearly co-localizes with shugoshin, PP2A, and cohesin at inner centromeres in meiosis II than in meiosis I. Morpholino-based depletion of SET from mouse oocytes caused some sister chromosomes to fail to separate in meiosis II [8]. Qi et al. reported similar findings [9]. In this case, SET depletion by RNAi did not detectably alter chromosome segregation in mouse oocytes, but overexpression of SET led to precocious sister separation in meiosis I. These results are broadly consistent with a model in which SET inhibits PP2A in meiosis II to allow Rec8 phosphorylation and cleavage by separase (Figure 1B).

SET is a member of a widely conserved family of proteins related to nucleosome assembly protein-1 (Nap1), which all form a distinctive “earmuff-like” structure. These include human and *Drosophila* SET, Vps75 in budding yeast, and the Nap1 proteins (e.g. six Nap1-like proteins in humans, Nap1 in *Drosophila*, and yNap1 in yeasts) [10], [11]. SET and Nap1 have both been extensively studied as histone chaperones; they can also associate with histone acetyltransferases (Vps75) and histone deacetylases (Nap1), and SET is a component of INHAT, an inhibitor of histone acetyltransferases [10]–[12]. It is notable that neither Chambon et al., nor Qi et al., formally showed that it is the ability of SET to inhibit PP2A activity that modulates cohesion. Therefore, a number of questions remain unanswered. Is SET really acting as a PP2A enzyme inhibitor? If so, does a similar mechanism exist in mitosis or in other organisms? Do the related Nap1 proteins play similar roles? The new study in this issue [3] expands on these points.

Moshkin et al. identified cohesin subunits in *Drosophila* Nap1 immunoprecipitates, and found that the genome binding sites of Nap1 resemble those of cohesin in ChIP-chip experiments [3]. Depletion or deletion of Nap1 caused increased chromosomal localization of PP2A, shugoshin/MEI-S332, and cohesin, and prevented the normal separation of chromosome arms in early mitosis. Based on this, and the movement of Nap1 into the nucleus in prophase, the ability of recombinant Nap1 to displace PP2A from cohesin complexes in vitro, and the ability of PP2A depletion or overexpression to reverse the cohesion defects caused by Nap1 depletion or overexpression, Moshkin et al. propose that Nap1 displaces PP2A from binding to cohesin. In this way, Nap1 increases cohesin phosphorylation and release by (presumably) the prophase pathway in mitosis, without necessarily inhibiting the enzyme activity of PP2A (Figure 1A).

Together, these three studies reveal that SET and Nap1 proteins regulate cohesion in meiosis and mitosis in more than one organism, and leave us with a variety of proposed ways in which the “guardian spirit” of cohesion (shugoshin-PP2A) can be relieved of its duties. It will now be interesting to determine if the different conclusions reached about the mechanism of SET and Nap1 action on PP2A reflect differences between the two proteins, between mice and flies, between meiosis and mitosis, or simply our currently incomplete understanding. Is counteracting PP2A action the only or main way in which these proteins regulate cohesion, or could their histone chaperone activity, or binding to histone-modifying enzymes, play a role? The future use of separation of function mutants of Nap1 and SET will likely help answer these questions. What normally restricts SET activity to meiosis II, and might cyclin A2–dependent kinases [13] contribute? Does SET function in mitosis? Does Nap1 play a role in meiosis, or influence centromeric cohesion in late mitosis? To what extent do tension-dependent shugoshin-PP2A relocation [5], [6] and PP2A inactivation [3], [8], [9] collaborate at centromeres? Because Nap1 can modulate gene expression, and cohesin itself may play a role in transcriptional regulation [1], [12], it will also be useful to exclude indirect effects on cell division.

Chromosome segregation defects cause a range of problems, including chromosome instability and aneuploidy in cancer and, if they occur in meiosis, infertility, miscarriage, and birth defects [14], [15]. Cohesin gene mutations in cancer [16] and loss of sister chromosome cohesion in aged oocytes [15], [17], [18] may underlie some of these defects. Further understanding of cohesion regulation, including the newfound contribution of Nap1 and SET, may enhance our ability to prevent and treat these conditions.
